# Relations Between Student Procrastination and Teaching Styles: Autonomy-Supportive and Controlling

**DOI:** 10.3389/fpsyg.2018.00809

**Published:** 2018-05-23

**Authors:** Nuria Codina, Rafael Valenzuela, Jose V. Pestana, Joan Gonzalez-Conde

**Affiliations:** ^1^Social Psychology and Quantitative Psychology, Universitat de Barcelona, Barcelona, Spain; ^2^Euncet Business School, Barcelona, Spain

**Keywords:** procrastination, Self-Determination Theory, controlling teaching behaviors, psychological needs, perceived competence

## Abstract

Procrastination is a complex problem that can be defined as delaying an intended course of action (despite anticipating adverse consequences). Even when some students have equivalent motivation and skill levels, they tend to procrastinate more frequently than others. Approaches that analyze whether contextual influences may prevent or promote dysregulation processes associated with procrastination are scarce. According to Self-Determination Theory, contextual influences can facilitate self-regulated motivation (e.g., autonomous pursuit of interests or personal goals), if teaching style is autonomy-supportive and guarantees the satisfaction of students’ basic psychological needs for perceived competence, autonomy, and relatedness. Contrariwise, school context can also impede the development of autonomous motivation if teachers frustrate the satisfaction of their students’ psychological needs by recurring to controlling teaching behaviors, such as controlling use of rewards, negative conditional regard, excessive personal control, or intimidation. The goal of the present study was to assess the relations between controlling and autonomy-supportive teaching behaviors, psychological needs satisfaction (of the needs for competence, autonomy, and relatedness), and four distinct measures of procrastination: general procrastination, decisional procrastination, procrastination linked to task avoidance, and pure procrastination. Data based on public university undergraduate students (*N* = 672) shows that controlling teaching behaviors are associated negatively with psychological needs satisfaction and positively with procrastination. Contrariwise, autonomy-supportive teaching behaviors are positively associated with psychological needs satisfaction and negatively with procrastination. The data obtained is useful for suggesting new lines of research to study the link between contextual influences and the prevention of academic procrastination in view of Self-Determination Theory. Also, our results suggest new pedagogical approaches where teachers can create contextual conditions that help to prevent or reduce procrastinating tendencies.

## Introduction

Procrastination is the delay in taking an intended course of action – despite anticipating adverse consequences. It is generally considered detrimental to subjective well-being, in addition to causing harm in the areas of physical, mental, economic and academic health (Klingsieck, 2013). These consequences have led to proposals for different ways of measuring procrastination and different kinds of intervention programs aimed at counteracting it Schouwenburg (2004) and Boldaji et al. (2015). Accordingly, research on the reasons for procrastination has been carried out from the perspectives of personality, education, clinical psychology and psychodynamics, though often unaccompanied by recommendations for intervention and, even when there are, these are planned from a specific perspective. In this sense, an important limitation is that the interventions do not integrate complementary knowledge stemming from other orientations (Klingsieck, 2013), and there is a lack of contrasted knowledge about the proposed therapeutic interventions. For their part, these interventions mainly focus on the learning of time management strategies or on cognitive approaches (Klingsieck, 2013), which is due in part to the poor systematization of analysis and intervention and the practically null assessment of the interventions carried out.

One aspect of this lack of systematic research into the different challenges posed by procrastination stands out in particular: the multifactorial nature of the causes and processes of ongoing procrastination (Steel, 2007). This multifactorial nature requires the development of a systematic body of knowledge that indicates relations between the various manifestations of procrastination and critical variables, strengthening the possibility of interpretation of these diverse findings. Such an approach could shed light on the reasons for procrastination, by pointing out its associations with specific variables (from middle range theories), hence informing about psychological processes that may counteract it.

In this respect, one of the central characteristics of procrastination – the lack of self-regulation – has been explained in motivational terms and more specifically in relation to self-determined motivation (Lee, 2005; Steel, 2007). According to the theory that posits this type of motivation, Self-Determination Theory (hereinafter, SDT), autonomous engagement in activities (tendency – by definition – opposed to procrastination) is linked to the development of quality motivation, which depends on people perceiving that their psychological needs are satisfied (Deci and Ryan, 2000). These needs are (Ryan, 1995): perceived autonomy (i.e., perceiving freedom to choose which activities to engage in and how to cope with them), perceived competence (i.e., perceiving that one can perform the activity well), and relatedness (i.e., feeling non-isolated and affectively close to others in the context of the activity). As regards the study of procrastination, SDT has antecedents that have shown associations between high levels of self-determined motivation and lower levels of procrastination (Lee, 2005). Likewise, it has been observed that procrastination may be associated with the fear of academic failure, with self-perceived competence having a possible impact on this association. In particular, when faced by fear of failure, people with high self-perceived competence have trust in their capacity to succeed and cope with the challenge, while those with low self-perceived competence respond negatively and avoid it Haghbin et al. (2012).

The above premises prompt us to ask in which ways context may be associated with procrastination and which aspects of individual experience are more closely linked to the processes of self-regulation responsible for inter-subject procrastination variations. In this respect, we explore two theoretically opposite teaching styles (in line with SDT): controlling teaching style – known as the dark side of motivation, and autonomy-supportive teaching style – referred to as the bright side of motivation (Haerens et al., 2015). By promoting psychological needs satisfaction, autonomy-supportive style engenders positive consequences for students on a personal level, such as better academic performance and greater well-being (Adie et al., 2008). On the other hand, controlling teaching style – by frustrating the satisfaction of psychological needs – can have adverse consequences for students, such as resistance to learning or not paying attention to teachers (De Meyer et al., 2016).

Recent research on controlling teaching style has identified four types of controlling behaviors (Castillo et al., 2014): controlling use of rewards (CUR, offering verbal or tangible rewards to get students involved in a task, complete it or reach a certain performance level: Deci et al., 1999); negative conditional regard (NCR, withdrawing affect or attention if, for example, a student does not achieve the expected results or does not display the attributes desired by parents or teachers: Assor et al., 2004); excessive personal control (EPC, for example, meddling in students’ private lives, i.e., in matters not directly linked to academic activities: Bartholomew et al., 2010); and intimidation (INT, use of verbal abuse such as shouting, threats, or humiliation: Bartholomew et al., 2010).

The above antecedents reveal the importance of conjointly investigating the links between these teaching behaviors of control and autonomy support, satisfaction of psychological needs and procrastinating behaviors among students. Specifically, we tested the following five hypotheses in this paper:

H_1_: Controlling teaching style is positively associated with student procrastination.
H_2_: Controlling teaching style is negatively associated with students’ psychological needs satisfaction.
H_3_: Autonomy-supportive teaching style is negatively associated with student procrastination.
H_4_: Autonomy-supportive teaching style is positively associated with students’ psychological needs satisfaction.
H_5_: Students’ psychological needs satisfaction is negatively associated with student procrastination.

## Materials and Methods

### Participants

The sample consisted of 675 undergraduate students on a daytime schedule at the University of Barcelona. The sampling method consisted of fixed quotas, each with a minimum of 15 participants. Quotas were established according to gender, year within curriculum, and field of knowledge. Participants were 407 women and 264 men (plus four participants who did not report their gender), coming from the fields of Arts and Humanities (*n* = 124), Natural Sciences (*n* = 84), Health Sciences (*n* = 190), Juridical and Social Sciences (*n* = 162), and Engineering (*n* = 115). The mean age was 19.81 years (*SD* = 2.26). After analyzing the database for missing values, three cases were discarded. Thus, the final sample analyzed in the results consisted of 672 students.

### Instruments

Data was collected using a total of seven instruments accompanied by the required demographics. The answer format was standardized to help students respond more efficiently and improve the quality of their answers. Specifically, all the items on each scale were rated from 1 to 5 on the same Likert-type scale (ranging from 1 = *Does not describe you at all* to 5 = *Very characteristic of you*).

The following instruments were used to analyze each variable.

#### Controlling Teaching Behavior

It was assessed using the Controlling Teaching Behaviors Scale, originally designed by Bartholomew et al. (2010, Controlling Coach Behaviors Scale) for a sports context and subsequently validated for Spanish population by Castillo et al. (2014). In the case of this study, the scale was adapted to fit the university academic context (based on Hein et al., 2015). Specifically, the expression “my coach” was replaced by “my teacher/my teachers” in the corresponding statements. This scale consists of 15 items and assesses four dimensions: CUR (four items), NCR (four items), INT (four items), and EPC (three items). Reliability of the subscales was acceptable (α_CUR_ = 0.746; α_NCR_ = 0.760; α_INT_ = 0.780; α_EPC_ = 0.529). Given that Cronbach’s alpha fell below the recommended level of 0.70 in the case of the EPC alpha, Nunnally’s (1967) criterion was taken into account to interpret this low value, thus allowing values between 0.50 and 0.60 and also taking into account that this low value could be explained by there being only three items, due to the sensitivity of alpha to the number of indicators (Güttler, 2009).

#### Teacher Support of Student Autonomy

The Autonomy Support Scale (Williams and Deci, 1996) was used, specifically the version validated by Nuñez et al. (2012) for Spanish university population. The scale consists of 15 items and assesses a single dimension, with acceptable reliability (α = 0.912).

#### Psychological Needs Satisfaction

This was assessed using the Scale of Psychological Needs Satisfaction in Education (Gillet et al., 2008), to be exact the Spanish version for the educational context developed by León et al. (2011). The scale consists of 15 items and assesses the satisfaction of the psychological needs for perceived competence (COM; five items), perceived autonomy (AUT; five items) and perceived relatedness (REL; five items). The reliability of its subscales was acceptable (α_COM_ = 0.812; α_AUT_ = 0.658; α_REL_ = 0.796), as determined by the alpha benchmark for factors with few indicators (α > 0.60; e.g., Malhotra and Birks, 2007) and its sensitivity to the number of indicators (Güttler, 2009).

*Procrastination.* In this research, procrastination was considered as both a general tendency and as specific to individuals, taking into account those instruments that better integrate the multi-faceted nature of procrastination (according to the analysis provided by Díaz-Morales et al., 2006). Thus, we used the General Procrastination Scale (GP; Lay, 1986), the Decisional Procrastination Questionnaire (DP; Mann, 1982, Unpublished), the Adult Inventory of Procrastination (AIP; McCown and Johnson, 1989) and the Pure Procrastination Scale (PP; Steel, 2010), consisting of 12 items stemming from the three previous scales, which has shown its potential – in comparison to the rest of procrastination measures – elsewhere (e.g., Steel, 2010; Svartdal and Steel, 2017). The Spanish translation by Díaz-Morales et al. (2006) was used in all cases and reliability of all these scales was acceptable (α_GP_ = 0.754; α_DP_ = 0.807; α_AIP_ = 0.853; α_PP_ = 0.832).

### Procedure

Data collection was carried out in paper format, during class time and in person. The answer sheets included all the scales, but the scales were presented in randomly counterbalanced order so as to avoid the effect of fatigue on the results. The instrument was applied during the first term of the 2016–2017 academic year, beginning 1 month after the start of the classes and ending 15 days before the final exams. This timing was important: it was intended to prevent contagion by outside variables such as extra academic work, tiredness, and class absenteeism (Dewitte and Schouwenburg, 2002). Data collection was carried out by members of the research team, previously trained in the application of the set of research instruments. With the consent of the teaching staff at the center and after presenting the research project, the students answered the instrument voluntarily; in fact, the students were only allowed to participate if they agreed to sign the informed consent. The time required to answer the questions in the set of instruments was less than an hour. Data confidentiality was guaranteed and the data was processed using R 3.4.3.

The ethical requirements of the Ethics Committee of the University of Barcelona were applied to the current study, which meant that additional approval for the research was not required since data obtained did not involve animal or clinical experimentation. Additionally, this study complies with the recommendations of the General Council of Spanish Psychological Associations (Consejo General de Colegios de Psicólogos) and also the Spanish Organic Law on Data Protection (15/1999: Jefatura del Estado, 1999).

## Results

Pearson’s product-moment correlations between variables of interest were calculated (**Table 1**). Means for controlling teaching behaviors yielded low scores on the aforementioned Likert scale. The lowest was 1.45 for Intimidation (INT: *SD* = 0.64) and the highest was 2.30 for NCR (*SD* = 0.86), while Controlling Use of Rewards (CUR: *M* = 1.67, *SD* = 0.70) and EPC (*M* = 1.79, *SD* = 0.72) yielded means within that range. With regard to Autonomy Support, its mean was 2.9 (AS: *SD* = 0.68). In the case of Psychological Needs Satisfaction, Autonomy had the lowest mean (AUT: *M* = 2.70, *SD* = 0.72), Perceived Relatedness had the highest (REL: *M* = 3.96; *SD* = 0.66), and Perceived Competence fell in between (COM: *M* = 3.67; *SD* = 0.71). Lastly, regarding procrastination measures, AIP had the lowest mean (AIP: *M* = 2.45, *SD* = 0.68) while GP had the highest (GP: *M* = 2.82, *SD* = 0.54). DP had a mean of 2.69 (DP: *SD* = 0.83) and PP had a mean of 2.72 (PP: *SD* = 0.64).

**Table 1:** Correlations between four controlling teaching behaviors, autonomy-supportive teacher style, students’ psychological needs satisfaction and four measures of procrastination.

	CUR	NCR	INT	EPC	AS	COM	AUT	REL	GP	DP	AIP	PP
CUR	–											
NCR	0.37^∗∗^	–										
INT	0.42^∗∗^	0.54^∗∗^	–									
EPC	0.37^∗∗^	0.46^∗∗^	0.53^∗∗^	–								
AS	0.09^∗^	–0.26^∗∗^	–0.30^∗∗^	–0.20^∗∗^	–							
COM	0.03	–0.18^∗∗^	–0.23^∗∗^	–0.22^∗∗^	0.43^∗∗^	–						
AUT	0.03	–0.20^∗∗^	–0.17^∗∗^	–0.22^∗∗^	0.47^∗∗^	0.45^∗∗^	–					
REL	–0.07	–0.13^∗∗^	–0.17^∗∗^	–0.09^∗^	0.23^∗∗^	0.45^∗∗^	0.22^∗∗^	–				
GP	0.06	0.06	0.09^∗^	0.07	–0.11^∗∗^	–0.24^∗∗^	–0.16^∗∗^	–0.05	–			
DP	0.10^∗^	0.08^∗^	0.13^∗∗^	0.15^∗∗^	–0.13^∗∗^	–0.26^∗∗^	–0.13^∗∗^	–0.06	0.58^∗∗^	–		
AIP	0.01	0.11^∗∗^	0.09^∗^	0.09^∗^	–0.11^∗∗^	–0.22^∗∗^	–0.07	–0.10^∗^	0.68^∗∗^	0.45^∗∗^	–	
PP	0.08^∗^	0.10^∗∗^	0.16^∗∗^	0.14^∗∗^	–0.10^∗∗^	–0.26^∗∗^	–0.11^∗∗^	–0.06	0.79^∗∗^	0.75^∗∗^	0.71^∗∗^	–
*M*	1.67	2.30	1.45	1.79	2.90	3.67	2.70	3.96	2.82	2.69	2.45	2.72
*SD*	0.70	0.86	0.64	0.72	0.68	0.71	0.72	0.66	0.48	0.83	0.68	0.64

*CUR, controlling use of rewards; NCR, negative conditional regard; INT, intimidation; EPC, excessive personal control; AS, autonomy support; COM, competence need satisfaction; AUT, autonomy need satisfaction; REL, relatedness need satisfaction; GP, general procrastination; DP, decisional procrastination; AIP, adult inventory of procrastination; PP, pure procrastination. ^∗^*p* < 0.05 level (two-tailed); ^∗∗^*p* < 0.01 (two-tailed). *N* = 672.*

Concerning correlations, perceived controlling teaching style and procrastination displayed associations in the hypothesized (positive) direction (H_1_). CUR was positively associated with DP (*r* = 0.10, *p* < 0.05) and PP (*r* = 0.08, *p* < 0.05). NCR was positively associated with DP (*r* = 0.08, *p* < 0.05), AIP (*r* = 0.11, *p* < 0.01), and PP (*r* = 0.10, *p* < 0.01). INT was associated with all four types of procrastination: GP (*r* = 0.09, *p* < 0.05), DP (*r* = 0.13, *p* < 0.01), AIP (*r* = 0.09, *p* < 0.05), and PP (*r* = 0.16, *p* < 0.01). Lastly, EPC was positively associated with DP (*r* = 0.15, *p* < 0.01), AIP (*r* = 0.09, *p* < 0.05), and PP (*r* = 0.14, *p* < 0.01).

Three out of four controlling teaching behaviors showed associations in the hypothesized (negative) direction (H_2_) with satisfaction of all three psychological needs. NCR was negatively associated with COM (*r* = -0.18, *p* < 0.01), AUT (*r* = -0.20, *p* < 0.01), and REL (*r* = -0.13, *p* < 0.01). INT was negatively associated with COM (*r* = -0.23, *p* < 0.01), AUT (*r* = -0.17, *p* < 0.01), and REL (*r* = -0.17, *p* < 0.01). EPC was negatively associated with COM (*r* = -0.22, *p* < 0.01), AUT (*r* = -0.22, *p* < 0.01), and REL (*r* = -0.09, *p* < 0.05). CUR, however, was not associated with psychological needs satisfaction.

As hypothesized (H_3_), autonomy support showed negative associations with all four measures of procrastination: GP (*r* = -0.11, *p* < 0.01), DP (*r* = -0.13, *p* < 0.01), AIP (*r* = -0.11, *p* < 0.01), and PP (*r* = -0.10, *p* < 0.01). Also, as hypothesized (H_4_), AS showed robust positive associations with satisfaction of all three psychological needs: COM (*r* = 0.43, *p* < 0.01), AUT (*r* = 0.47, *p* < 0.01), and REL (*r* = 0.23, *p* < 0.01).

Finally, associations between student procrastination and the satisfaction of their psychological needs for competence, autonomy and relatedness were observed in the hypothesized (negative) direction (H_5_). Perceived competence showed negative associations with all four measures of procrastination: GP (*r* = -0.24, *p* < 0.01), DP (*r* = -0.26, *p* < 0.01), AIP (*r* = -0.22, *p* < 0.01), and PP (*r* = -0.26, *p* < 0.01). Perceived autonomy showed negative associations with GP, DP, and PP: GP (*r* = -0.16, *p* < 0.01), DP (*r* = -0.13, *p* < 0.01), PP (*r* = -0.11, *p* < 0.01). Contrariwise, the correlation between Perceived autonomy and AIP was not statistically significant. For its part, the third psychological need, perceived relatedness, showed one negative association with AIP (*r* = -0.10, *p* < 0.05).

Not only were our hypotheses confirmed but we also noted, as regards the relation between the two teaching styles, that three out of four controlling teaching behaviors were negatively associated with autonomy support: NCR (*r* = -0.27, *p* < 0.001), INT (*r* = -0.30, *p* < 0.01), EPC (*r* = -0.20, *p* < 0.01). However, CURs showed a positive association with autonomy support (*r* = 0.09, *p* < 0.05).

The role of perceived competence (COM) as a moderator in the relations between teacher style and procrastination was assessed using moderated multiple regression analysis. A total of 16 models were calculated, one for each pair of predictor variables (i.e., COM-CUR, COM-NCR, COM-INT, and COM-EPC) and criteria variables (procrastination: GP, DP, AIP, and PP). This procedure implied a series of steps. Firstly, coefficients of predictor variables were centered. Secondly, each pair of centered predictor variables was multiplied to obtain its interaction term. Thirdly, each regression model included corresponding centered variables and their interaction terms as predictors. **Table 2** displays those moderated multiple regression models in which the direct effect of teacher style and the interaction effect were both statistically significant.

**Table 2:** Moderated multiple regression models explaining procrastination.

Model	*B*	β	*SE*	*t*	*p*	*R^2^*	Adjusted *R^2^*	*F*	*p(F)*
EPC → DP						0.09	0.09	22.06	<0.001
Intercept	2.71		0.03	86.66	<0.001				
EPCc	0.13	0.11	0.04	2.89	<0.01				
COMc	–0.31	–0.26	0.05	–6.78	<0.001				
EPCc^∗^COMc	0.14	0.10	0.05	2.76	<0.01				
INT → DP						0.09	0.08	21.25	<0.001
Intercept	2.71		0.03	86.76	<0.001				
INTc	0.13	0.10	0.05	2.54	<0.05				
COMc	–0.32	–0.27	0.05	–6.95	<0.001				
INTc^∗^COMc	0.15	0.11	0.05	2.93	<0.01				
INT → PP						0.09	0.09	22.63	<0.001
Intercept	2.73		0.02	114.05	<0.001				
INTc	0.13	0.13	0.04	3.34	<0.001				
COMc	–0.24	–0.26	0.04	–6.77	<0.001				
INTc^∗^COMc	0.12	0.12	0.04	3.05	<0.01				

*EPCc, excessive personal control (centered); COMc, perceived competence (centered); INTc, intimidation (centered); DP, decisional procrastination; PP, pure procrastination.*

As **Table 2** shows, centered perceived competence (COMc) was a moderator in three of these moderated regression models. Specifically, competence had a statistically significant moderating role in the regression of DP relative to EPCc (adjusted *R*^2^ = 0.09; *F* = 22.06, *p* < 0.001), of DP relative to INTc (adjusted *R*^2^ = 0.08; *F* = 21.25, *p* < 0.001), and of PP relative to INTc (adjusted *R*^2^ = 0.09; *F* = 22.63, *p* < 0.001). The findings of the models can be described as follows. In the moderated regression model of DP relative to EPCc (i.e., centered EPC) and COMc (i.e., centered COM), both EPCc and COMc had statistically significant direct effects (respectively, β = 0.11 and β = -0.26). Estimates showed that COMc had a greater direct effect on DP than EPCc. Interaction term had a positive value (β = 0.10), thus indicating that the clearer a given student’s perception that their psychological need for competence had been fulfilled, the more positive the effect of EPC on DP.

Similar effects were observed in the moderated regression of DP relative to INTc (i.e., INT centered) and COMc. INTc had a positive direct effect on DP (β = 0.10) and COMc had a negative direct effect on DP (β = -0.27). Again, COMc had a greater effect than controlling teaching style (in this case, INTc). Interaction term had a positive value (β = 0.11), indicating that the greater the perceived competence of a student, the more positive the effect of intimidation on DP.

Lastly, the moderated regression model of PP relative to INTc and COMc showed that, while INTc had a positive direct effect on PP (β = 0.13), COMc had a negative direct effect (β = -0.26), with the latter showing an absolute effect stronger than the former. Interaction term was positive (β = 0.12), indicating that the greater the perceived competence of a student, the greater the positive effect of intimidation on PP.

In sum, presented findings mainly support the hypotheses of the present study. Firstly, student procrastination was positively associated with controlling teaching behaviors and negatively associated with autonomy-supportive teaching behaviors and psychological needs satisfaction. Secondly, psychological needs satisfaction was positively associated with autonomy-supportive teaching behaviors and negatively associated with controlling teaching behaviors. Finally, these interrelations between teaching styles, psychological needs satisfaction, and student procrastination were also indicated through moderated regression analyses: satisfaction of the need for competence (perceived competence) was a moderator variable affecting the relationship between controlling teaching behaviors and student procrastination; specifically, as students feel more competent, intimidation and EPC have a worse direct effect on decisional and PP.

## Discussion

In the quest to learn more about the social context of procrastination, teaching style is a variable that deserves particular consideration. In this study, 13 of the 16 possible associations between controlling teaching behaviors and student procrastination were significant and positive, although their effect sizes were rather small. To sum up our analyses, we briefly highlight the ways in which each of the four studied measures of procrastination correlated with analyzed variables.

• General procrastination showed negative associations with perceived autonomy support and satisfaction of the needs for competence and autonomy. Contrarily, GP correlated positively with perceptions of controlling teaching behaviors involving intimidation. And, furthermore, higher GP scores were related to lower perceived competence.
• Procrastination linked to task avoidance (AIP) showed negative associations with autonomy support and the satisfaction of the needs for competence and relatedness. Contrarily, it showed positive associations with NCR, intimidation and EPC. Direct effect values lay in between those yielded by GP and DP.
• Pure procrastination correlated negatively with autonomy support and perceived competence and autonomy. Contrarily, PP correlated positively with all four controlling teaching behaviors. Even though direct effect values were low, they were mostly highly significant.
• Decisional procrastination showed negative associations with autonomy support and perceived competence and autonomy. DP also showed positive associations with all four controlling teaching behaviors. Direct effect values were low, but not as low as in the case of GP; the two most significant associations between DP and controlling teaching style concerned the behaviors comprising intimidation and EPC.
• Lastly, perceived competence moderated the relations between EPC and DP, between intimidation and DP, and between intimidation and PP. Specifically, the more students perceived themselves as competent, the more intimidation and EPC predicted inter-subject procrastination variations.

Put differently, this study offers some support for this argument: autonomy-supportive teaching style (reported by students) is associated negatively with procrastination and positively (and somewhat robustly) with the satisfaction of the needs for competence and autonomy.

Based on SDT predictions, satisfaction of psychological needs favors optimal conditions for the development of autonomous motivation, engagement and self-regulation (Deci and Ryan, 2000; Ryan and Deci, 2000) and, thus, is associated negatively with procrastination, given that the latter depends on dysregulation processes, which clash with a high degree of engagement (Lee, 2005). In line with the postulates of SDT, autonomy-supportive teaching style may contribute to the promotion of motivation and regulation conditions that counteract procrastination, such as the satisfaction of psychological needs, also enhancing students’ possibilities of autonomous engagement in learning. Contrariwise, controlling teaching behaviors were negatively associated with satisfaction of all three psychological needs, and the satisfaction of these needs was negatively associated with procrastination measures. Given these findings, it seems that students who report that their teacher used any controlling teaching behaviors on them are more likely to procrastinate, possibly, given that their psychological needs are more likely to be frustrated.

Our research enriches scientific knowledge about procrastination by incorporating SDT. This incorporation highlights how student procrastination is connected with teaching styles. Particularly, it is observed that higher levels of procrastination are associated with controlling teaching behaviors such as intimidation and EPC. Even more, through SDT, our results have showed that predictive effects of controlling teaching style on procrastination variations are worsened by higher levels of competence need satisfaction.

Regarding future studies, they should try to answer if potential adverse effects of controlling teaching style on procrastination can be differentiated between teachers rewarding aspects strictly pertaining to academic task performance and teachers exercising intimidation or trying to be controlling on extra-academic levels. Also, further research could benefit from person-centered approaches, which could address the question whether students with higher and lower perceived competence report different amounts of controlling teaching behaviors and if in fact this is objectively true or if it is their subjective interpretation.

With this research, SDT’s explanatory potential is broadened to a promissory subject area like procrastination behaviors, additionally, in a more applied sense, the conjoint analysis of procrastination and SDT could help professionals design interventions aimed at providing learners with the right conditions to help them avoid and counteract procrastination. In particular, this research suggests new pedagogical approaches in which teachers can create contextual conditions that prevent or reduce procrastinating tendencies.

## Author Contributions

NC conceived and designed the research for this paper. She was also responsible for drafting the work and revising it critically for important intellectual content. RV contributed with the analysis of the results from the standpoint of Self-Determination Theory. JP gave support to the design of the research and also the data analysis. JG-C co-coordinated data collection and prepared the documents required for gathering information. He also participated during all the phases of the data analysis and writing of this paper.

## Conflict of Interest Statement

The authors declare that the research was conducted in the absence of any commercial or financial relationships that could be construed as a potential conflict of interest.
